# The “Chameleon Ant” *Colobopsis imitans* Adapts Its Mimetic Appearance to Local Model Species Across the Mediterranean Basin (Hymenoptera: Formicidae)

**DOI:** 10.1002/ece3.72674

**Published:** 2025-12-26

**Authors:** Herbert C. Wagner, Sándor Csősz

**Affiliations:** ^1^ Institute of Biology University of Graz Graz Austria; ^2^ HUN‐REN‐ELTE‐MTM Integrative Ecology Research Group Budapest Hungary; ^3^ Department of Systematic Zoology and Ecology, Institute of Biology ELTE‐Eötvös Loránd University Budapest Hungary

**Keywords:** Batesian mimicry, *Camponotus lateralis*, *Colobopsis truncata*, color microevolution, *Crematogaster ionia*, *Crematogaster scutellaris*, discriminant‐function analysis, *Dolichoderus quadripunctatus*, nest‐centroid clustering

## Abstract

Mimicry, where an organism (the mimic) convergently evolves traits resembling another (the model), is one of the most compelling phenomena in evolutionary biology. Despite ants frequently serving as models for Batesian mimics in other arthropod taxa, mimicry among ants is still underexplored. Rapid mimetic adaptations may superficially suggest a pathway to speciation; therefore, a thorough exploration of the phenomenon requires multivariate analyses. Consequently, we collected morphometric data from *Colobopsis* samples across the Mediterranean Basin and the Caucasus, documented color patterns of mimics and local models, and performed unsupervised multivariate analyses to examine evolutionary dynamics in the Mediterranean *Colobopsis* mimicry system. Our central questions were whether adaptive color changes in regional mimic populations reflect distinct evolutionary lineages leading to speciation or represent intraspecific responses to local environmental pressures, that is, adjustment to locally available models. We sought morphometric discontinuities in morphospace that might indicate the existence of distinct lineages among mimic populations with different color schemes. Biogeographic analyses show that *Colobopsis imitans* Schifani et al., 2022 replaces *Colobopsis truncata* (Spinola, 1808) throughout southern Europe and displays remarkably versatile region‐specific mimetic visual adaptations to local model species—hence the title analogy; this species is a true chameleon, in a biogeographical sense. Our research presents a scenario where mimicry‐driven microevolutionary adaptations can produce significant phenotypic diversity without leading to speciation.

## Graphical abstract

We collected morphometric data from *Colobopsis* samples across the Mediterranean Basin and the Caucasus, documented color patterns of these mimics and their local models, and performed unsupervised multivariate analyses. Morphometric data suggest a scenario where mimicry‐driven microevolutionary adaptations can produce significant phenotypic diversity without leading to speciation. Biogeographic analyses show that *Colobopsis imitans* displays remarkably versatile region‐specific mimetic visual adaptations to local model species.

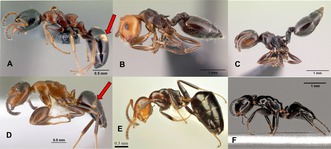

## 1 Introduction

Mimicry, a phenomenon in which one organism (the mimic) evolves a close resemblance to another (the model), is a compelling example of natural selection (Poulton 1890; Wickler 1968; Kikuchi and Pfennig 2013; Quicke 2017; McLean et al. 2024). In Batesian mimicry, harmless prey species mimic models with higher defensive capabilities, toxicity, inedibility, or evasiveness (Bates 1861; Wickler 1968; Páez et al. 2021). Resembling the size, shape, color, or behavior of the models (Rubio et al. 2013; Pekár 2022; McLean et al. 2024) reduces predation pressure (Palmgren et al. 1937; Taniguchi et al. 2005; Nelson 2012; Ramesh et al. 2016). Although ants are among the most frequently mimicked models by various arthropod groups such as spiders, beetles, bugs, and non‐ant hymenopterans (McIver and Stonedahl 1993), documented cases of ants visually mimicking other ant species are rare, with only a little bit more than 20 known species (Wagner et al. 2025). While in most studies the mimicry is only briefly mentioned, there are a few detailed investigations (e.g., Ito et al. 2004; Gallego Ropero and Feitosa 2014; Pekár et al. 2017; Zettel et al. 2018). This scarcity makes inter‐ant mimicry particularly intriguing from an evolutionary perspective.

Several examples illustrate the geographic variation in ant‐to‐ant mimicry. The *Camponotini* species *Camponotus lateralis* (Olivier, 1792) exhibits remarkable regional color adaptations that correspond to local and unpalatable *Crematogaster* species (H. C. Wagner 2025; Wagner et al. 2025): Italian populations display often blackish bodies with reddish heads, mirroring the situation in *Crematogaster scutellaris* (Olivier, 1792); most Balkan populations resemble *Crematogaster schmidti* (Mayr, 1853) with reddish heads and mesosomas; while southeastern Greek populations are uniformly brownish to blackish like *Crematogaster ionia* Forel, 1911 (Kraker and Wagner 2025). An intriguing, similar case is the two color‐morphs of *Camponotus guanchus* Santschi, 1908 from the Canary Islands, which exhibit two distinct color morphs that align with the sympatric, congruently colored subspecies of *Cr. alluaudi* Emery, 1893, with no intermediate forms detected (Pérez‐Delgado and Wagner 2024). In Australia, the subspecies *Camponotus suffusus bendigensis* Forel, 1902 mimics the painful‐stinging *Myrmecia fulvipes* Roger, 1861, in whose range it is embedded (Merrill and Elgar 2000). While these three examples represent cases of distinct phenotype differences at an intraspecific level, a color adaptation has recently been proposed to be an initial step toward speciation (Schifani et al. 2022). However, distinguishing true speciation events that cross evolutionary divergence thresholds (Seifert 2020a) from intraspecific color variations requires comprehensive multivariate analyses.

West‐Palearctic *Colobopsis* species like *Colobopsis truncata* (Spinola, 1808) are strictly arboricolous on broad‐leaved trees (Forel 1892; Brun 1924; Eidmann 1926; Schifani et al. 2022; Giannetti et al. 2024). Only a few Central European ant species use similar microhabitats (Czechowski et al. 2012; Schuler 2015; Seifert 2018). Among them, *Dolichoderus quadripunctatus* (Linnaeus, 1771) is the only one resembling *Co. truncata* in terms of color and size (Forel 1874; Lebas et al. 2016; H. C. Wagner 2019; García 2020; Schifani et al. 2022). The colonies of these two species are often located close to each other (Stitz 1939). In Vienna (Austria), at least 36% of the trees with *Co. truncata* colonies were also occupied by *Do. quadripunctatus* (*n* = 110); in the Italian mainland, the value was 40% (*n* = 35; Schifani et al. 2022). Rarely, *Co. truncata* follows the trails of *Do. quadripunctatus* (Kutter 1977; H. C. Wagner 2019). The phenotypical and ecological similarity between the two species, in combination with the sharp taste of *Do. quadripunctatus*, has led to the hypothesis of Batesian mimicry in *Co. truncata* (Forel 1874; H. C. Wagner 2019; Schifani et al. 2022).

In the Mediterranean, Collingwood (1993) found *Colobopsis* on trees with *Crematogaster* and recognized the color and size similarity between workers of both genera. As he considered *Crematogaster* distasteful, he speculated that mimicry in *Colobopsis* reduces predation by birds. Batesian mimicry was also suggested recently by Schifani et al. (2022), who investigated the color, ecology, and association between *Colobopsis* and *Cr. scutellaris*. Moreover, they postulated mimicry‐driven speciation in *Colobopsis* by using integrative taxonomic approaches to identify *Colobopsis imitans* Schifani et al., 2022 as a distinct evolutionary lineage. This species was described to differ from its sister species *Co. truncata* in both color and morphology while the new species mimicked the red‐headed *Cr. scutellaris*. Initially, it was described as a West‐Mediterranean species occurring in northwestern Africa, Iberia, and Sicily (Schifani et al. 2022, 2024), but our understanding of *Co. imitans* continues to develop as new populations are discovered and analyzed.

Our discovery in 2024 of fully blackish *Colobopsis* minor workers following trails of *Cr. ionia* and *Cr*. cf. *ionia* sp. 1 sensu Salata et al. (2020) on Crete (Wagner et al. 2025) provided the catalyst for the present study. The distinct coloration of these Cretan specimens, along with their shift in model selection compared to mainland European *Co. truncata* and the previously described *Co. imitans* populations, raises fundamental questions about the relationship between mimicry and speciation in this species complex.

This study addresses whether Mediterranean *Colobopsis* populations are adapted to mimic the color of sympatric models, and whether the observed shifts in model selection represent (1) distinct evolutionary lineages warranting species‐level recognition, or (2) intraspecific regional adaptations resulting from fast color microevolution. Using multivariate morphometric analyses of *Colobopsis* material collected across the West‐Palearctic region—including Italy, Central Europe, the Balkans, Crete, Anatolia, Cyprus, Crimea, and the Caucasus—we sought to identify morphometric discontinuities that might indicate distinct evolutionary lineages among populations exhibiting different color schemes.

Our research provides insights into the wide Mediterranean distribution of *Co. imitans* and underscores that its populations have undergone microevolutionary adaptations to sympatric local model species rather than representing distinct species.

## 2 Materials and Methods

### 2.1 Materials

Morphometric data of 105 minor workers from the dataset of Schifani et al. (2022) were used for the analyses, and all these ants were reinvestigated to collect values of two further morphometric characters (see Section 2); 103 further minor workers were newly investigated. The morphometric analyses focused only on minor workers, as too few major workers and gynes for the application of morphometric statistics were available.

*Colobopsis truncata*: a total of 40 nest samples with 100 workers were investigated morphometrically. They were from Austria (11), Bulgaria (1), Croatia (5), Greece mainland (2), Hungary (4), Italy mainland (13), Montenegro (1), North Macedonia (2), and Romania (1).

*Colobopsis imitans*: a total of 46 nest samples with 108 workers were investigated morphometrically. They were from Anatolia (4), Azerbaijan (4), Crete (12), Crimea (1), Cyprus (1), Greece mainland (1), Morocco (2), North Macedonia (1), Samos (2), Sicily (17), and Spain (1).

### 2.2 Morphometrics

If available, three minor workers per sample were mounted. Workers were dried for at least 48 h (see Seifert 2002 regarding shrinkage due to moisture loss). Morphometric data were collected using a Leica MZ16 A high‐performance stereomicroscope with magnifications of ×80 to ×296. Workers were positioned on a pin‐holding stage permitting spatial adjustment in all directions. A cross‐scaled ocular micrometer with 120 graduation marks was used. Its measuring line was kept vertically to avoid parallax error (Seifert 2002). A combination of a Fiberoptic L 150 light, equipped with two flexible light ducts, and a Leica KL 1500 LCD coaxial polarized light were used.

All morphometric characters given in Schifani et al. (2022) were measured. The definition of the character ML originally read (Schifani et al. 2022, 6): “Diagonal length of the alitrunk in profile. Measured in lateral view from the anteriormost point of anterior pronotal slope to the caudalmost point of the lateral metapleural lobe.” Since this definition contradicted the morphometric data of the Supplement material of Schifani et al. (2022), it had to be transformed. The mesosoma length ML was in fact measured from the anteriormost point to the posteriormost end. Two further characters were additionally added: depth of metanotal groove (MGr) (Seifert 2019) and depth of dorsal propodeal groove (PrGr). All bilateral characters were measured from both sides, and arithmetic means were calculated (Table 1).

**TABLE 1:** Acronyms and definitions of morphometric characters.

Acronym	Definition
CL	Maximum cephalic length in median line; head is carefully tilted to position with true maximum; excavation of occiput reduces CL.
CW	Maximum head width including compound eyes. The largest distance between profiles of the two compound eyes in full‐face view.
CS	Head (cephalic) size; the arithmetic mean of CL and CW, used as a less variable indicator of body size (Seifert 2018).
dAN	Minimum distance of the inner margins of antennal socket rings in dorsofrontal view (see figure 271 in Seifert 2018, 400).
EL	Eye length. Maximum diameter of the compound eye.
HTL	Hind‐tibia length. Measured from the distalmost point of the tibia to the proximal end where the tibia is narrowest in profile (see figure 385 in Seifert 2018, 401).
MGr	Depth of metanotal groove. In lateral view, measured perpendicular from a tangent touching the dorsalmost points of mesonotum and propodeum to the deepest point of groove (Figure 1).
ML	Mesosoma length from the anteriormost point of pronotum to the posteriormost end of metapleuron.
MW	Maximum width of pronotum.
NOL	Petiole length; measured in lateral view, from the center of the petiolar spiracle to the posterior profile.
PeSH	Petiole height measured in lateral view from the center of petiolar spiracle to top of the crest.
PeW	Petiole width. Maximum width of petiole in dorsal view.
PreOc	Preocular distance. Use a cross‐scaled ocular micrometer and adjust the head to the measuring position of CL. Frontal measuring‐point: median clypeal margin; caudal measuring‐point: reference line between the frontalmost border of the two compound eyes.
PrGr	Depth of dorsal propodeal groove. In lateral view, measured perpendicular from a tangent touching the uppermost points of frontal and caudal portions of propodeum (Figure 1).
SL	Scape length. Maximum straight‐line scape‐length excluding the articular condyle.

**FIGURE 1:**
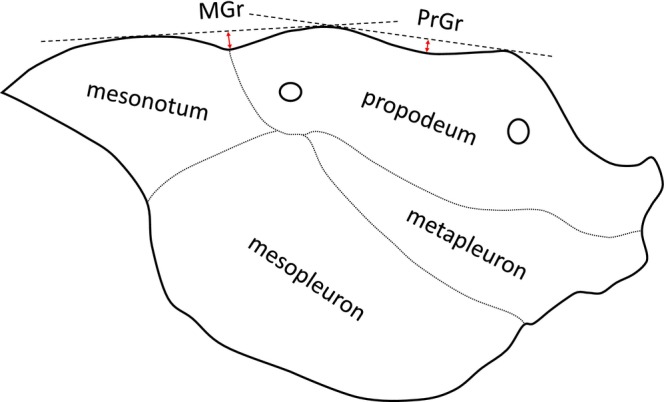
Visual representation of the morphometric characters MGr (metanotal groove) and PrGr (dorsal propodeal groove). The figure shows the mesosoma (without pronotum) in lateral view (anterior to the left).

Nest centroid (NC) clustering (Seifert et al. 2014; Csősz and Fisher 2015, 2016) was used as an unsupervised approach to establish morphological species hypotheses, using R version 4.4.2 (R Core Team 2024) and the packages MASS, ecodist, cluster, plyr, stringr, and scatterplot3d (Ligges and Maechler 2003; Goslee and Urban 2007; Wickham 2009; Maechler et al. 2013; Ripley et al. 2013). Raw morphometric data were used to establish a hierarchical NC‐clustering to outline species hypotheses. Subsequently, two partitioning methods, “part.kmeans” and “part.hclust,” were applied to estimate the cluster number based on a statistical threshold and to assign cases into clusters (Supporting Information: File S1). Samples with discordant results were treated as wild cards and were assigned to groups through confirmative linear discriminant analyses (LDA) using all morphometric data at the worker level, utilizing the “stepwise selection” method in SPSS Statistics v21 (IBM, USA).

Morphometric characters in *Colobopsis* can be positively allometric, such as MW (i.e., larger individuals have a wider mesosoma relative to CS), or negatively allometric, such as SL (i.e., larger individuals have shorter scapes relative to CS). Allometric effects do not influence results of NC clustering and LDAs, but they do influence principal‐component analyses (PCA; B. Seifert, personal communication). This is because PCAs extract axes of the maximum total variance, which in ants are often affected by allometric variation. To avoid this effect, calculating allometric corrections is often an essential data‐processing step for PCAs. As standard for both species, removal of allometric variance after Seifert (2008) was calculated assuming all minor workers to have a cephalic size of CS = 0.9 mm. The variables *k* and *d* of linear functions y=k·x+d of morphometric data were calculated using the scatter plot (XY) tool in MS Excel for each character and species separately. Arithmetic means of *Co. truncata* and *Co. imitans* were substituted into the formula:

$$y/x_{0.9}=\frac{y/x}{k\cdot \mathrm{CS}+d}\cdot \left(k\cdot 0.9+d\right)$$

The resulting functions to correct the allometric variance of characters for the PCA were as follows:

$${\text{CL/CW}}_{0.9}=\frac{\mathrm{CL}/\mathrm{CW}}{-0.1223\cdot \mathrm{CS}+1.2597}\cdot 1.1496$$

$${\text{dAN/CS}}_{0.9}=\frac{\mathrm{dAN}/\mathrm{CS}}{0.2193\cdot \mathrm{CS}+0.1921}\cdot 0.3895$$

$${\text{EL/CS}}_{0.9}=\frac{\mathrm{EL}/\mathrm{CS}}{-0.0539\cdot \mathrm{CS}+0.3693}\cdot 0.3207$$

$${\text{HTL/CS}}_{0.9}=\frac{\mathrm{HTL}/\mathrm{CS}}{-0.1818\cdot \mathrm{CS}+1.0808}\cdot 0.9172$$

$${\text{MGr/CS}}_{0.9}=\frac{\mathrm{MGr}/\mathrm{CS}}{-0.0255\cdot \mathrm{CS}+0.0430}\cdot 0.0200$$

$${\text{ML/CS}}_{0.9}=\frac{\mathrm{ML}/\mathrm{CS}}{0.0220\cdot \mathrm{CS}+1.4492}\cdot 1.4690$$

$${\text{MW/CS}}_{0.9}=\frac{\mathrm{MW}/\mathrm{CS}}{0.1484\cdot \mathrm{CS}+0.5459}\cdot 0.6795$$

$${\text{NOL/CS}}_{0.9}=\frac{\mathrm{NOL}/\mathrm{CS}}{-0.0127\cdot \mathrm{CS}+0.1488}\cdot 0.1374$$

$${\text{PeSH/CS}}_{0.9}=\frac{\text{PeSH}/\mathrm{CS}}{0.0769\cdot \mathrm{CS}+0.1790}\cdot 0.2482$$

$${\text{PeW/CS}}_{0.9}=\frac{\mathrm{PeW}/\mathrm{CS}}{0.1097\cdot \mathrm{CS}+0.2360}\cdot 0.3347$$

$${\text{PreOc/CL}}_{0.9}=\frac{\text{PreOc}/\mathrm{CL}}{0.1009\cdot \mathrm{CS}+0.4478}\cdot 0.5386$$

$${\text{PrGr/CS}}_{0.9}=\frac{\text{PrGr}/\mathrm{CS}}{0.0250\cdot \mathrm{CS}+0.0011}\cdot 0.0236$$

$${\text{SL/CS}}_{0.9}=\frac{\mathrm{SL}/\mathrm{CS}}{-0.3655\cdot \mathrm{CS}+1.1843}\cdot 0.8553$$

A PCA with a reduced character set as suggested by the LDA was applied on the correlation matrix using PAST 4.13 (Hammer et al. 2001).

Two‐sided, type‐2 *t* tests (Student 1908) were calculated to assess differences in indices and CS between workers of *Co. imitans* and *Co. truncata*.

### 2.3 Geographic Distribution of Color Schemes

We hypothesize that *Do. quadripunctatus* serves as the primary model for *truncata*‐like‐colored *Colobopsis* populations (Forel 1874; H. C. Wagner 2019; Schifani et al. 2022) and *Cr. scutellaris* as the model for *Co. imitans* in the West Mediterranean (Schifani et al. 2022, 2024). Dark‐colored taxa of the *Cr. scutellaris* complex, *Cr. ionia*, *Cr*. cf. *ionia* sp. 1, and *Cr*. cf. *ionia* sensu Demetriou et al. (2024), hereafter collectively referred to as *Cr. ionia* s. l., are hypothesized to be models for blackish *Colobopsis* minor workers. Since *Colobopsis* workers of both species also follow trails of *Cr. schmidti* (Mayr, 1853) and the head coloration of *Co. truncata* is often lighter than in *Do. quadripunctatus* (thus intermediate between *Cr. schmidti* and *Do. quadripunctatus*), *Cr. schmidti* may also serve as a model.

To assess a potential nonrandomness in the distribution patterns of *Colobopsis* color morphs and their sympatric models, we constructed a model presence–absence matrix based on zoogeographic data (see distribution map) and applied Fisher's exact tests (R. A. Fisher 1922, 1925) via the online tool https://www.quantpsy.org/fisher/fisher.htm. In cases of multiple tests, Bonferroni–Holm corrections (Holm 1979) were applied.

The distribution data of *Do. quadripunctatus* is primarily based on Map 8 in Czechowski et al. (2012, 63) and the verbal description provided by Seifert (2018). This information has been updated with recent findings from southern Europe, Anatolia, and the Caucasus (Paknia et al. 2010; Lebas et al. 2016; Borowiec and Salata 2018, 2022a, 2022b; Schifani and Alicata 2018; Bračko 2019; Cabanillas et al. 2019; Kiran and Karaman 2020; Borowiec et al. 2021, 2022, 2024).

The distribution information for *Cr. scutellaris* follows Lebas et al. (2016), supplemented with data from Zimmermann (1935), Bračko (2006, 2023), Seifert (2018), Gouraud (2025), and own collecting activity around the Adriatic Sea. Utilizable images from iNaturalist (https://www.inaturalist.org/) were also considered regarding the presence of the species in North Africa and eastern France.

Distribution data for *Cr. ionia* s. l. were collected from various literature (Forel 1913; Vonshak and Ionescu‐Hirsch 2009; Kiran and Karaman 2012, 2020; Bračko et al. 2016; Borowiec and Salata 2017, 2018, 2020, 2024; Salata and Borowiec 2019; Salata et al. 2020; Borowiec et al. 2021, 2024; Lapeva‐Gjonova and Borowiec 2022; Demetriou et al. 2024).

The *Colobopsis* data used for distribution analyses were exclusively sourced from material that was morphometrically analyzed by Schifani et al. (2022, 2024) and in the frame of the present study.

The distribution map was created using QGIS 3.32 (https://qgis.org/).

## 3 Results

### 3.1 Morphometrics

**FIGURE 2:**
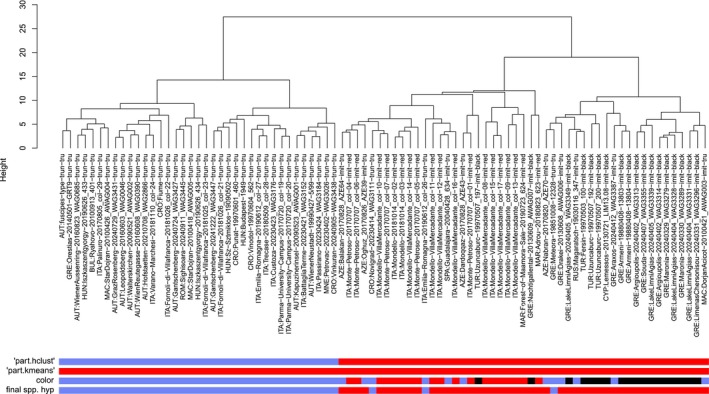
Dendrogram comparing nest‐centroid clustering (Ward method) of morphometric raw data of *Colobopsis* (singletons included) with algorithms “part‐hclust” and “kmeans.” Color information is given: Blue = *truncata*‐like color morph; red = red‐headed color morph; black = blackish color morph.

The NC clustering revealed two primary clusters (morphometric data in Supporting Information: File S2). Both the NC‐Ward and NC‐part.hclust exploratory data analyses indicated the presence of two clusters with a classification error of 3.5% determined by the controlling linear discriminant function. NC‐part.kmeans clustered all samples as a single group (Figure 2).

**FIGURE 3:**
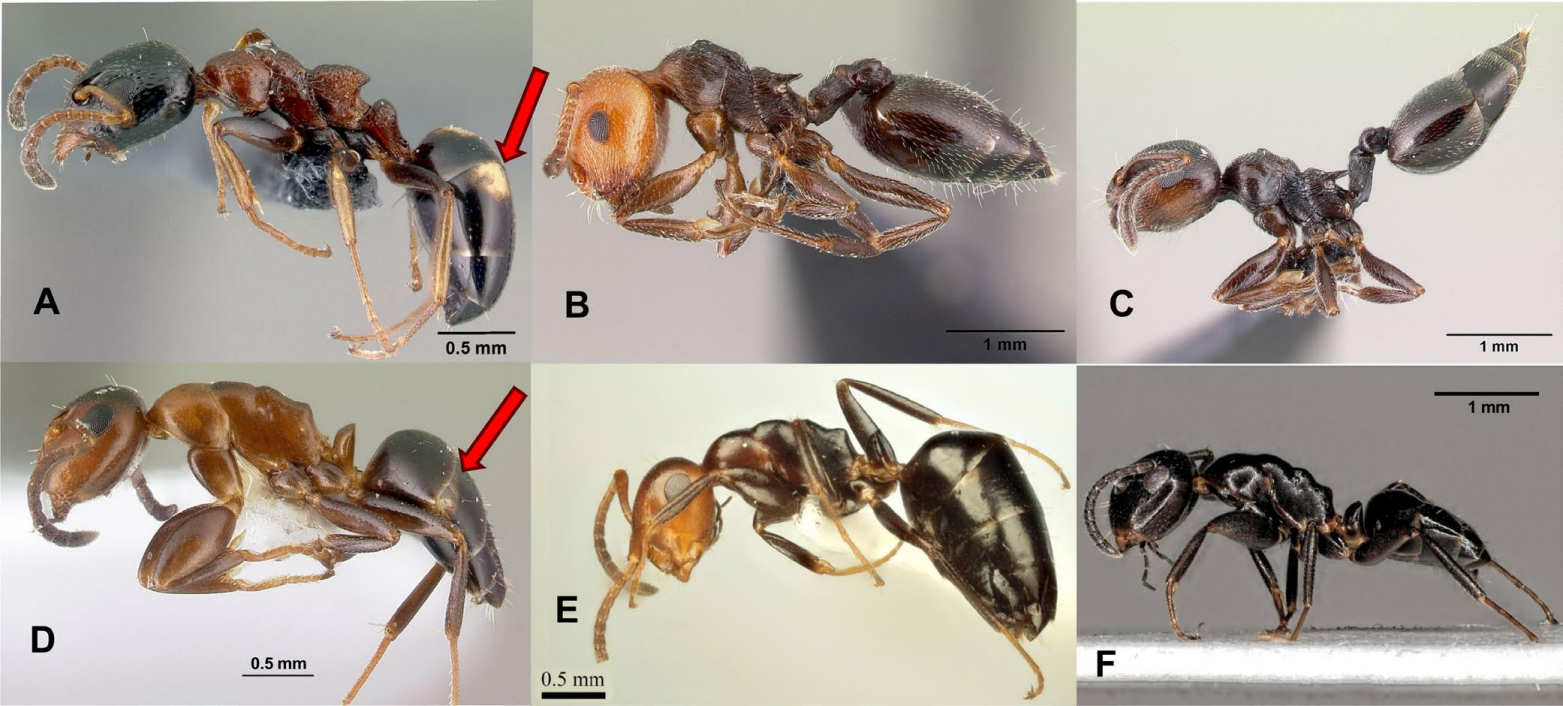
Comparison of three model species (A: *Dolichoderus quadripunctatus* ; B: *Crematogaster scutellaris* ; C: *Cr. ionia*) with three color morphs of the mimetic genus (D: *Colobopsis truncata*; E: red‐headed color morph of *Co. imitans*; F: blackish color morph of *Co. imitans*). Pictures of the models are placed above their mimics. *Dolichoderus quadripunctatus* and *Colobopsis truncata* have whitish dots on the second gastral tergite (red arrows). Photos: A: E. Prado (casent0179916, AntWeb, B. L. Fisher 2025); B: E. Prado (casent0179890, AntWeb); C: E. Prado (casent0179888, AntWeb); E. Prado (casent0179881, AntWeb); E: E. Schifani (antweb1041481, AntWeb); F: R. Schultz.

**FIGURE 4:**
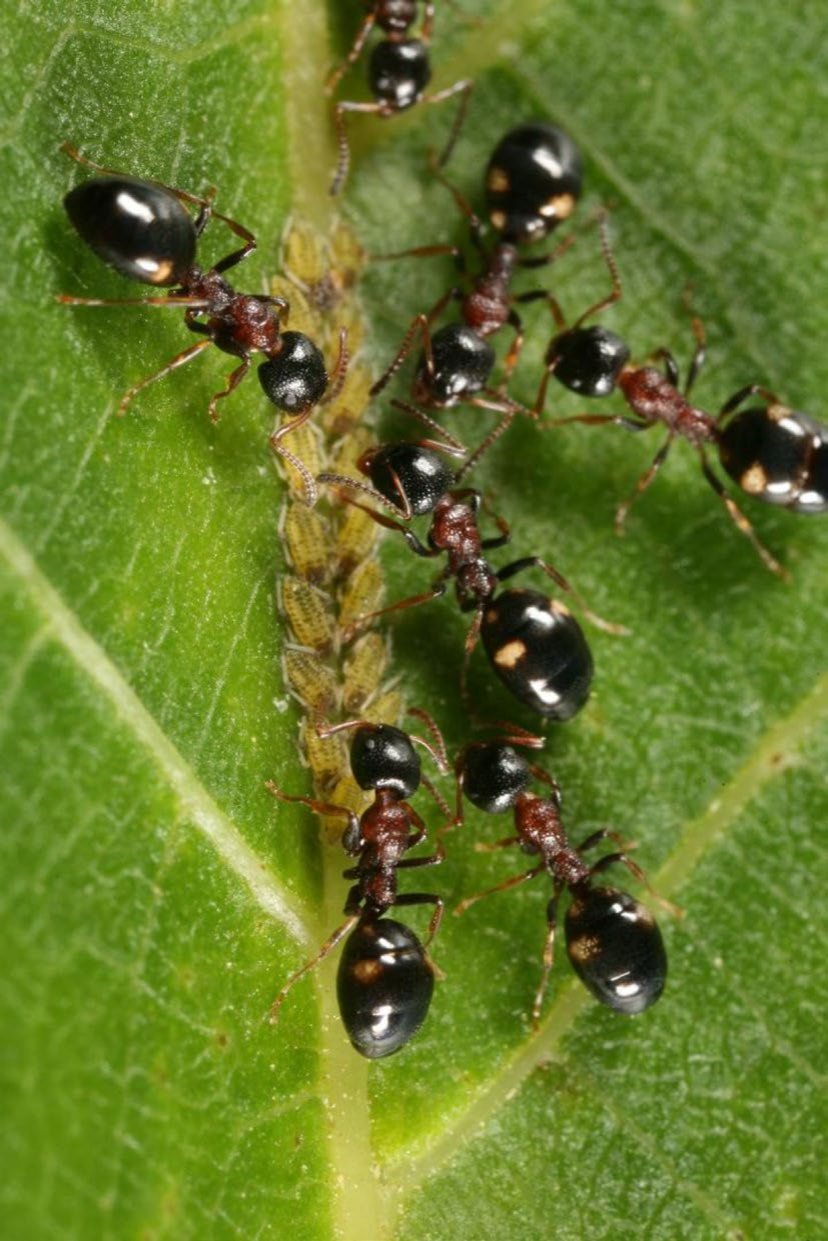
Workers of *Dolichoderus quadripunctatus* have four whitish dots on the gaster—this aposematic signal is mimicked by both species of the *Colobopsis truncata* complex. Photo: G. Csóka.

**FIGURE 5:**
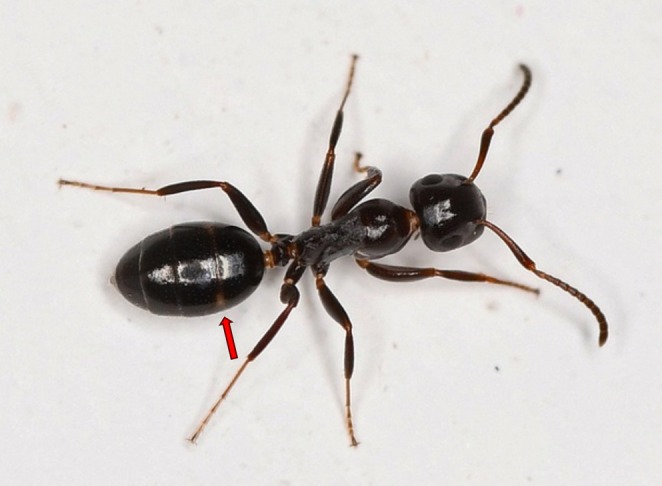
A blackish minor worker of *Colobopsis imitans* from Crete having rudimentary whitish dots on the second gastral tergite (red arrow), although *Dolichoderus quadripunctatus* is not native to Crete. Photo: F. Samaritakis.

While *Co. truncata* exhibits only one color morph, *Co. imitans* displays three distinct color morphs:

1. The *truncata*‐like color morph: this morph closely resembles the color scheme of *Co. truncata*: characterized by mesosoma and appendages from reddish to blackish, head at least slightly darker than the mesosoma. The dorsum of head appears darker than the genae, clypeus, and mandibles, while the gaster is blackish (Figure 3). White stripes or dots on the second gastral tergite, similar to those observed in *Do. quadripunctatus* (Figures 3 and 4), are frequently present: We detected at least remnants of them in 86% of *Co. truncata* minor‐workers (*n* = 58) and 46% of *truncata*‐like *Co. imitans* minor‐workers (*n* = 13).
2. Red‐headed color morph: as originally described, head, or the head and anterior part of the mesosoma, typically uniformly reddish; while remainder of the body appears dark‐brown to blackish (Figure 3). White stripes or dots on the second gastral tergite were present in 10% of the examined workers (Schifani et al. 2022).
3. Blackish color morph: this color morph exhibits entirely blackish coloration (Figure 3) with remnants of white stripes or dots present in 50% of 44 workers examined (Figure 5).

**FIGURE 6:**
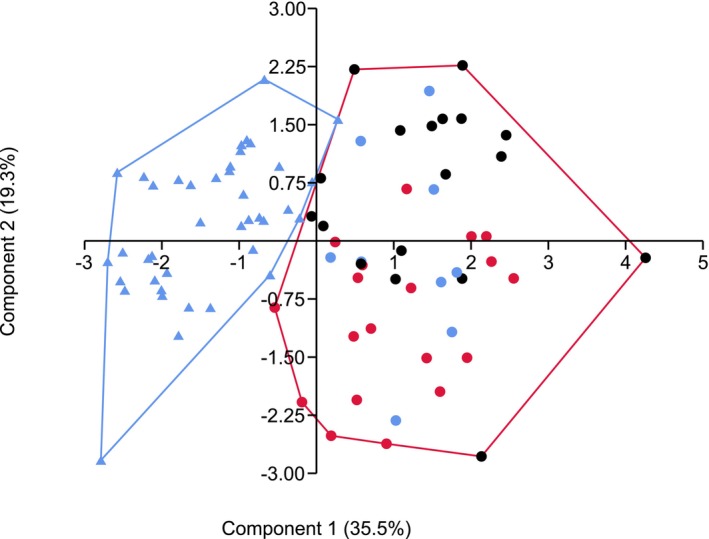
Principal‐component analyses using 7 RAV‐corrected indices of all *Colobopsis* nest‐means (singletons included). Triangles represent *Co. truncata* samples, circles *Co. imitans* samples, colors the *truncata*‐like (blue), red‐headed (red), and blackish (black) color morphs.

A PCA with seven selected RAV‐corrected indices (CL/CW_0.9_, HTL/CS_0.9_, MGr/CS_0.9_, NOL/CS_0.9_, PeSH/CS_0.9_, PreOc/CS_0.9_, PrGr/CS_0.9_) of *Co. truncata* and *Co. imitans* nest‐means, including singletons, shows a tiny overlap (Figure 6): One sample of *Co. truncata* from Greece is placed at the margin of the *Co. imitans* polygon.

The discriminant 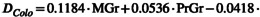 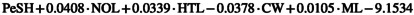 separates minor workers of *Co. imitans* from *Co. truncata*. Workers of *Co. imitans* have values > 0 (error rate 5.6% in 108 workers and 2.2% in 46 nest means) and workers of *Co. truncata* < 0 (error 2.0% in 100 workers and 0.0% in 40 nest means).

Nine of 14 morphometric characters are significantly different between *Co. imitans* and *Co. truncata* (Table 2). While the *truncata*‐like and the red‐headed color morphs of *Co. imitans* turn out to be identical concerning their morphometrics, the blackish color morph shows differences: Eye size in the blackish color morph is with EL/CS = 0.331 ± 0.007 (minimum = 0.318, maximum = 0.345) highly significantly larger than in *Co. truncata* (*p* < 0.001), but in the red‐headed color morph with EL/CS = 0.311 ± 0.010 (minimum = 0.290, maximum = 0.335) highly significantly smaller than in *Co. truncata* (*p* < 0.001). Counting all color morphs of *Co. imitans* together, the differences to *Co. truncata* cancel each other out (*p* = 0.1814).

**TABLE 2:** Mean of morphometric indices and CS calculated for *Colobopsis truncata* and *Colobopsis imitans* based on individuals (raw data) in μm.

Character	*Co. truncata* (*n* = 100)	*p*	*Co. imitans* (*n* = 108)
CS	893 ± 58 [736, 1042]	0.0516	878 ± 49 [725, 1025]
CL/CW	1.144 ± 0.019 [1.106, 1.189]	**< 0.0001**	1.158 ± 0.021 [1.113, 1.219]
dAN/CS	0.390 ± 0.016 [0.348, 0.426]	**0.0004**	0.383 ± 0.014 [0.342, 0.431]
EL/CS	0.323 ± 0.007 [0.304, 0.339]	0.1814	0.321 ± 0.012 [0.290, 0.345]
MGr/CS	0.017 ± 0.004 [0.000, 0.025]	**< 0.0001**	0.024 ± 0.005 [0.011, 0.043]
HTL/CS	0.903 ± 0.022 [0.845, 0.947]	**< 0.0001**	0.936 ± 0.022 [0.888, 0.986]
ML/CS	1.458 ± 0.023 [1.410, 1.526]	**< 0.0001**	1.479 ± 0.029 [1.395, 1.543]
MW/CS	0.676 ± 0.018 [0.639, 0.727]	0.4511	0.678 ± 0.019 [0.628, 0.724]
NOL/CS	0.133 ± 0.010 [0.108, 0.157]	**< 0.0001**	0.142 ± 0.010 [0.117, 0.169]
PeSH/CS	0.253 ± 0.018 [0.203, 0.293]	**< 0.0001**	0.242 ± 0.017 [0.192, 0.284]
PreOc/CL	0.537 ± 0.010 [0.506, 0.570]	0.9508	0.537 ± 0.014 [0.503, 0.571]
PeW/CS	0.336 ± 0.018 [0.301, 0.395]	0.0678	0.331 ± 0.016 [0.268, 0.372]
PrGr/CS	0.019 ± 0.008 [0.000, 0.040]	**< 0.0001**	0.027 ± 0.006 [0.009, 0.048]
SL/CS	0.851 ± 0.028 [0.796, 0.931]	**< 0.0001**	0.869 ± 0.027 [0.798, 0.932]

*Note:* The upper row in fields of columns 2 and 4 gives arithmetic mean ± standard deviation; the lower row, in square brackets, the lower and upper extremes. Significant differences (*p*; *t*‐test, 2 sides, type 2) after Bonferroni–Holm correction (Holm 1979) are highlighted in bold.

**FIGURE 7:**
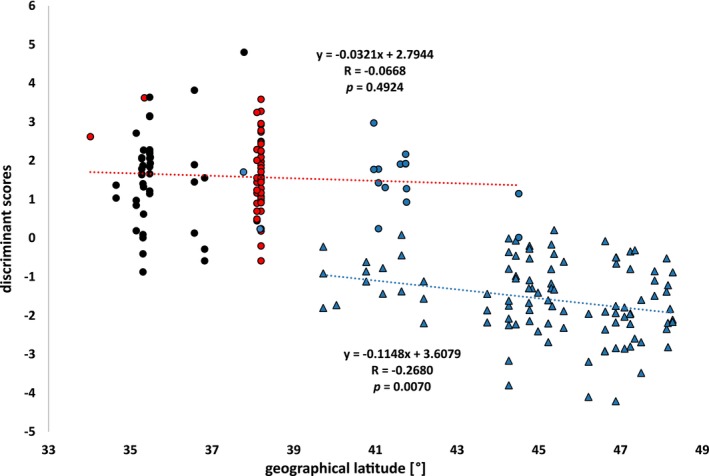
Relation between discriminant scores of the discriminant D_Colo_ and latitude. Circles represent *Co. imitans* workers, triangles *Co. truncata* workers, colors the *truncata*‐like (blue), red‐headed (red), and blackish (black) color morph. There is a negative correlation in *Co. truncata*, but no correlation in *Co. imitans*.

There is a significant negative correlation of the discriminant scores of D_Colo_ and geographical latitude in *Co. truncata* (*R* = −0.2680, *p* = 0.0070, Figure 7), showing a convergence of morphometric similarity in the sympatric zone, but no such correlation in *Co. imitans* (*R* = −0.0668, *p* = 0.4924).

### 3.2 Geographic Distribution of Color Schemes

Among putative model species, *Do. quadripunctatus* only is sympatric with *Co. truncata* at 13 sites (39%), *Do. quadripunctatus* with *Cr. scutellaris* at 14 sites (42%), *Do. quadripunctatus* with *Cr. schmidti* at five sites (15%), and only *Cr. scutellaris* on the Balearic Islands (3%; *n* = 33; Table 3). *Do. quadripunctatus* plus *Cr. schmidti* are sympatric with the *truncata*‐like color morph of *Co. imitans* at six sites (67%), *Do. quadripunctatus*, *Cr. ionia* with *Cr. schmidti* at two sites (22%), and *Do. quadripunctatus* with *Cr. scutellaris* at one site (11%). *Co. truncata* and the *truncata*‐like color morph of *Co. imitans* together are significantly more often with *Do. quadripunctatus* and *Cr. schmidti* than the red‐headed and the blackish color morphs (*p* < 0.0001 and 0.0295, respectively).

**TABLE 3:** Numbers of sites with sympatric occurrence of *Colobopsis truncata* and the three color morphs of *Colobopsis imitans* with their putative models.

Putative models	*Dolichoderus quadripunctatus*		*Crematogaster scutellaris*		*Crematogaster schmidti*		*Crematogaster ionia* s. l.	
Presence	Yes	No	Yes	No	Yes	No	Yes	No
*Co. truncata*	32	1	15	18	5	28	2	31
*Co. imitans*, *truncata*‐like color morph	9	0	1	8	8	1	2	7
*Co. imitans*, red‐headed color morph	0	8	8	0	0	8	0	8
*Co. imitans*, blackish color morph	1	9	0	10	1	9	10	0
Sum of mimics	41	1	8	0	13	29	10	0
Sum of nonmimics	1	17	16	36	1	17	4	46
*p* comparing sum of mimics and nonmimics	**< 0.0001**		**0.0003**		**0.0295**		**< 0.0001**	

*Note:* Significant differences after Bonferroni–Holm correction (Holm 1979) are highlighted in bold.

At all eight sites of the red‐headed color morph of *Co. imitans*, *Cr. scutellaris* is the only sympatric model species (100%). This color morph is significantly more often associated with *Cr. scutellaris* than *Co. truncata*, as well as the *truncata*‐like and the blackish color morphs of *Co. imitans* together (*p* < 0.0001).

At nine sites of the blackish color morph of *Co. imitans*, only *Cr. ionia* s. l. is present (90%); at one site (Samos, Nachtigallental), *Cr. ionia* is most common, but also *Cr. schmidti* and *Do. quadripunctatus* occur sympatrically. This color morph is significantly more often associated with *Cr. ionia* s. l. than *Co. truncata*, as well as the *truncata*‐like and the red‐headed color morphs of *Co. imitans* together (*p* < 0.0001).

*Dolichoderus quadripunctatus* is distributed from Iberia to Siberia. It regularly goes up to north between 47° and 52° N (Czechowski et al. 2012; Seifert 2018). In the south, it is present in the northern half of Iberia (Cabanillas et al. 2019), in France, on Corsica (Lebas et al. 2016), in mainland Italy (Schifani 2022), the whole Balkan mainland (Czechowski et al. 2012), on some Greek islands (like Lesvos, Samos, Kos) (Borowiec and Salata 2018, 2022a, 2022b; Borowiec et al. 2021, 2022, 2024), in western and northern Anatolia (Kiran and Karaman 2020), the Caucasus (Bračko 2019), and NW Iran (Paknia et al. 2010, Figure 8). It lacks in southern Iberia (Czechowski et al. 2012), NW Africa (Lebas et al. 2016; Schifani 2022), on the Balearic Islands (Eidmann 1926; Menozzi 1926; Ceballos 1956; Cabanillas et al. 2019), Sardinia (Schifani et al. 2021), in southern Anatolia (Kiran and Karaman 2012, 2020), and on Cyprus (Demetriou et al. 2024). On Sicily, apart from a single record on the southeast of the island, it also lacks (Schifani and Alicata 2018). It was not mentioned in the monograph of Cretan ants (Salata et al. 2020), but there is a single record from Rethymno city (Borowiec and Salata 2022a). Since the species was not found in near‐natural biotopes on Crete (Wagner et al. 2025), we interpret it as introduced. Isolated records in Scandinavia (Douwes et al. 2012), Siberia, and Central Asia (Czechowski et al. 2012; Seifert 2018) are not considered in our distribution map, since their large geographic distance to the next *Colobopsis* records makes them meaningless for the content of our study.

**FIGURE 8:**
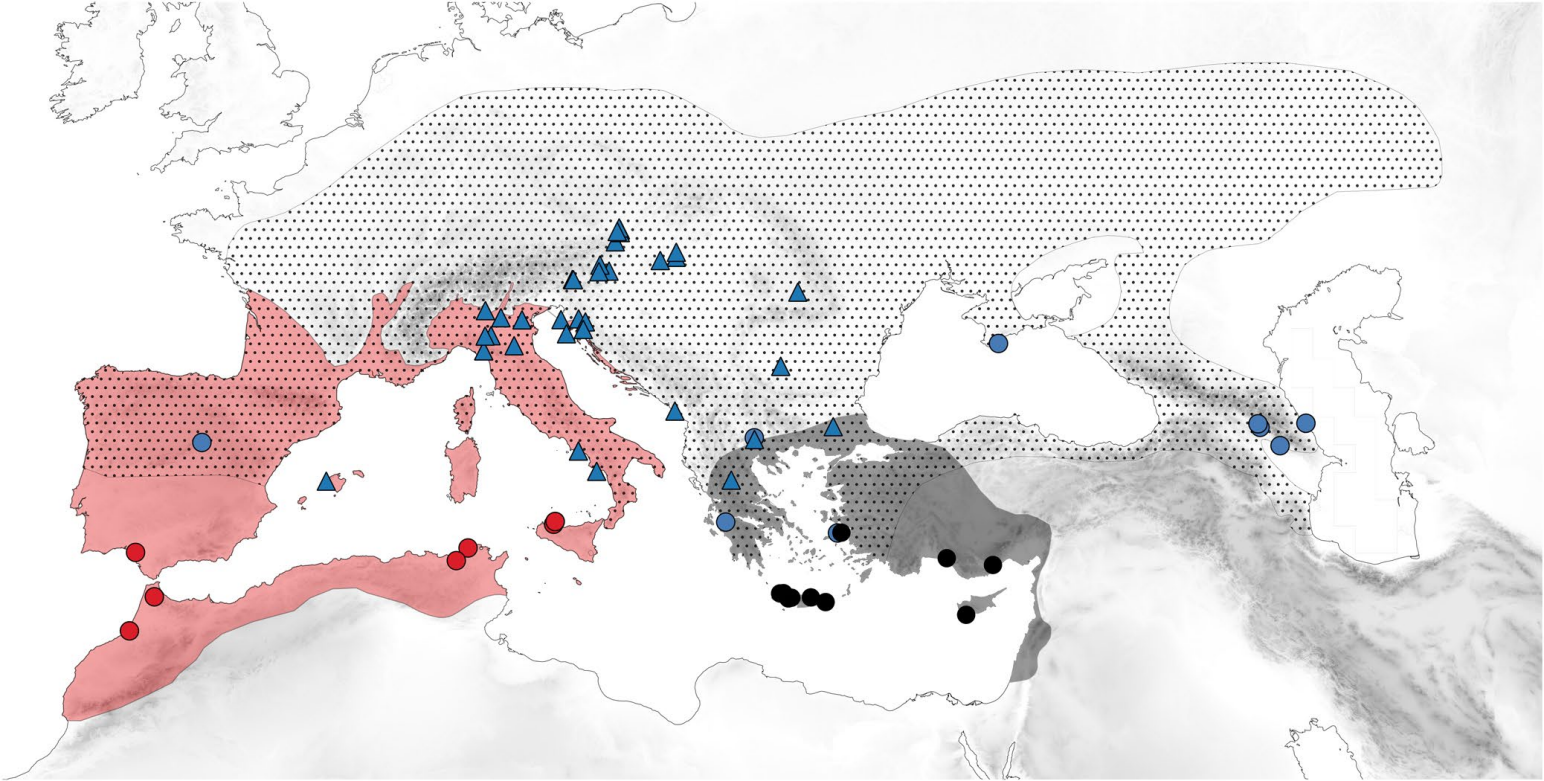
Geographic distribution of the two West‐Palearctic *Colobopsis* species and their three primary model species: *Dolichoderus quadripunctatus* (dotted area), *Crematogaster scutellaris* (reddish area), and *Crematogaster ionia* s. l. (blackish area); *Colobopsis truncata* (blue triangles), *Colobopsis imitans* of the *truncata*‐like (blue dots), the red‐headed (red dots), and the blackish color morph (black dots).

*Crematogaster scutellaris* is a West‐Mediterranean species occurring in NW Africa, Iberia, on the Balearic Islands, in S France (Gouraud 2025), Italy mainland, on Sardinia, Corsica (Casevitz‐Weulersse 1990), Sicily (Lebas et al. 2016; Seifert 2018), in Istria, Dalmatia (Zimmermann 1935), and on most Croatian islands (Zimmermann 1931; Bračko 2006, Figure 8). It is lacking in Slovenia (Bračko 2023). Seifert's (2018, 178) southeastern‐most record is not from Montenegro but from Dalmatia. The southeastern distribution boundary along the Dalmatian mainland coast is at the latitude of Island Hvar.

*Crematogaster ionia* s. l. occurs in the southernmost regions of the Balkan mainland (Bračko et al. 2016; Borowiec and Salata 2017; Salata and Borowiec 2019; Lapeva‐Gjonova and Borowiec 2022), on most or all Greek islands (Forel 1913; Borowiec and Salata 2018; Salata and Borowiec 2019; Salata et al. 2020; Borowiec et al. 2021, 2024), in the southwestern half of Anatolia (Forel 1913; Kiran and Karaman 2012, 2020), on Cyprus (Demetriou et al. 2024), and along the Mediterranean to the southeast until Israel (Forel 1913; Vonshak and Ionescu‐Hirsch 2009; Borowiec and Salata 2020, Figure 8). The record from Osijek (Croatia), cited in Bračko (2006), is to “almost 100% … a false record” (G. Bračko in litt. 2024).

## 4 Discussion

Comprehensive multivariate analyses of linear morphometric markers identified two distinct clusters representing the two species *Co. truncata* and *Co. imitans*. We did not detect further taxonomically significant clusters within the morphometric data; hence, our findings support the taxonomic classifications established by Schifani et al. (2022). Some of today's biologists might argue at this point that molecular‐genetic data would have been necessary to justify species delimitation. However, there is a great tradition in Central European myrmecology for delimitating species based solely on morphometric data (e.g., Seifert 1988a, 1988b, 2000, 2002, 2020b, 2021; Csősz and Seifert 2003; Seifert and Schultz 2009). The number of synonyms produced by doing so turns out to be tiny. Morphometry is reproducible (Csősz et al. 2021), and special statistical approaches have been established to analyze these data unsupervised (Seifert et al. 2014; Csősz and Fisher 2015, 2016). The success of the morphometric approach, compared to mitochondrial DNA, for example, likely reflects the strong correlation between morphology and nuclear DNA (Schlick‐Steiner et al. 2010; Seifert 2018, 2020a).

We discovered versatile mimetic color adaptations in *Co. imitans*, reflecting those of the local model species across the Mediterranean region. While *Co. truncata* exhibits no region‐specific differences across its range, *Co. imitans* mimics three primary local model species. The consistent color traits of populations of *Co. truncata* across its range may be influenced by conservative model‐species selection; the main and, in many regions, only model is *Do. quadripunctatus* across the entire distributional range of the mimetic species. In contrast, *Co. imitans* exhibits remarkable spatial color adaptation, which may be attributed to its wide distribution in fragmented areas of Mediterranean peninsulas and islands, where a variety of local species suitable for modeling are found.

### 4.1 Interspecific Character Convergence in Sympatric *Colobopsis* Populations

This study assessed whether the divergence of discriminant scores, a measure of morphological differentiation, was associated with latitude, which serves as a proxy for geographic distance between *Co. truncata* and *Co. imitans*. We observed increasing morphological convergence in sympatric populations (e.g., mainland Greece) in morphospace. This contradicts character displacement (Brown and Wilson 1956), a mechanism that is a marker and booster of reproductive isolation between closely related sympatric species (Stroud et al. 2024), as we would expect to see increasing morphological distance in sympatric areas.

Although sympatry of both *Colobopsis* species has been confirmed at only a few sites, and the relevant evolutionary time scales remain unclear, we propose several potential explanations.

The most plausible explanation is that reduced morphological divergence in sympatric areas may indicate (unidirectional) interspecific gene flow. Hybridization is widespread in nature (Mallet et al. 1998; Baack and Rieseberg 2007; Weyna et al. 2022) and particularly frequent in ants (Seifert et al. 2010; Steiner et al. 2011; Purcell et al. 2016; Pamilo and Kulmuni 2022). Hybridization occurs more frequently between closely related species (Seifert 1999; Cordonnier et al. 2019), especially in taxa that have undergone rapid and recent adaptive radiations (Gourbière and Mallet 2010; Abbott et al. 2013).

Co‐occurring populations of different *Colobopsis* species are subject to similar environmental constraints that favor phenotypes enhancing local fitness, often by mimicking common sympatric model species. These unidirectional competitive pressures can drive phenotypic evolution through character convergence when models are shared, or, on the contrary, through disruption when different models are used.

### 4.2 Mimetic Color Adaptations to Model Species

Although *Colobopsis* species across the Mediterranean display distinct local color schemes, multivariate analyses of linear morphometric data did not support this differentiation pattern. Instead, clear similarities in coloration with sympatrically occurring model species emerged. The primary model of *Co. truncata*, and of the *truncata*‐like color morph in its sister species *Co. imitans*, is the widely distributed *Do. quadripunctatus* . Workers of both genera frequently forage on the same trees (Forel 1874; Stitz 1939; Goetsch 1950; Schifani et al. 2022), rarely follow the same trails (Kutter 1977; H. C. Wagner 2019), and exhibit similar body sizes and colors (Forel 1874; H. C. Wagner 2019; García 2020; Schifani et al. 2022). While various European Camponotini ants are frequently consumed by birds (e.g., Rey 1910; Escherich 1917; Eidmann 1929; Conrads 1962; T. Wagner 1993; Hódar 1998; Michalek and Krištín 2009; Seifert 2009; del Val et al. 2018; Goffová et al. 2022) and lizards (Bombi and Bologna 2002; H. C. Wagner 2025; Wagner et al. 2025), reports about myrmecophagy of *Do. quadripunctatus* —despite the widespread occurrence and frequency of this model species—are very rare (Csiki 1905). Moreover, in our preliminary observations, an Italian wall lizard ( *Podarcis sicula* ) ingested *Ca. lateralis* (H. C. Wagner 2025) and *Co. truncata*, but spat out and subsequently avoided *Do. quadripunctatus* . Hence, we consider the aposematic signal of *Do. quadripunctatus* to be honest (Figure 4).

*Colobopsis imitans* exhibits region‐specific color patterns across its geographic range. The *truncata*‐like color morph mimics *Do. quadripunctatus* , the red‐headed color morph is associated with *Cr. scutellaris*, and the blackish color morph imitates *Cr. ionia* s. l. Our analysis supports the hypothesis that *Cr. schmidti* serves as a comodel for *Co. truncata* and the *truncata*‐like color morph of *Co. imitans* in terms of adaptive mimicry (Table 3). This is in line with the observation that *Colobopsis* workers often possess lighter head coloration compared to *Do. quadripunctatus*, resembling *Cr. schmidti*. Quantitative color measurements (see Schifani et al. 2022; Pekár et al. 2022; Kraker and Wagner 2025) will be crucial for elucidating the role of *Cr. schmidti* as a comodel in this Mediterranean mimicry system, especially for the *truncata*‐like color morph of *Co. imitans*, which was frequently found syntopically with *Cr. schmidti*.

To conclude, while the color patterns of West‐Palearctic *Colobopsis* minor workers differ intraspecifically in dependence on the sympatric models, they match across species boundaries if the model is the same. These results imply that colors may result from microevolutionary adaptations, younger than species divergence. It is not new for ant taxonomy that color is no indicator for species affiliation (Seifert 2018, 2019, 2025; Csősz et al. 2022), but, interestingly, in mimetic species, it is an indicator for models occurring sympatrically (Merrill and Elgar 2000; Schifani et al. 2022; Pérez‐Delgado and Wagner 2024; Kraker and Wagner 2025).

### 4.3 Local Mimetic Adaptation Mechanism Versus Speciation Process

Mimetic adaptation is a crucial mechanism in microevolution and promotes geographic color variation—usually at the intraspecific level (Mallet et al. 1998; The Heliconius Genome Consortium 2012; Jones et al. 2013; Edmunds and Reader 2014). When differences in mimetic color patterns are observed among incipient species, usually additional factors like geographic isolation or sexual selection may act as decisive factors leading to speciation (Jiggins 2008). However, mimetic color adaptation has recently been proposed as a starting point in speciation processes (Schifani et al. 2022). The impact of mimicry‐related color changes on visual appearance, and their interactions with heterospecifics is crucial for understanding the evolutionary history of the Mediterranean *Colobopsis* lineages and contributes to our knowledge of the mechanisms underlying evolutionary diversity and the processes shaping phenotypic outcomes.

Insects display diverse body colors and patterns that are specific to different species, populations, sexes, and can also change throughout their life stages (Hartmann et al. 2019; Khan 2020). Color tends to evolve more rapidly than morphology, often in response to climate changes (Haque et al. 2024), as single mutations can substantially alter insect pigmentation (Futahashi and Osanai‐Futahashi 2021). It is also known that mimetic color patterns in butterflies can result from phenotypic plasticity (Shimajiri and Otaki 2022).

Although our data indicate that all color variants of *Co. imitans* belong to the same species, we cannot fully exclude that these stable color variants represent initial steps in speciation processes. One can speculate that intermediate‐colored workers may have lower adaptive advantages than pure‐colored varieties, potentially reducing gene flow between these color variants. In Lepidoptera, ecological speciation due to assortative mating based on mimetic color traits has been documented (Jiggins 2008; Chamberlain et al. 2009). However, as long as color patterns have no meaning for assortative mating (as it is probably the case in *Co. imitans*), they are less likely to enhance speciation processes.

Several Camponotini mimic species exhibit intraspecific color morphs:

1.
*Camponotus lateralis* : all three color morphs utilize different models (Kraker and Wagner 2025) and belong to the same species (Seifert 2019). Color‐mixed nests and intermediate individuals occur.
2.
*Camponotus guanchus* : the two color morphs belong to the same species (Cagniant and Espadaler 1993). Although intermediate‐colored individuals have not been detected (Pérez‐Delgado and Wagner 2024), preliminary mitochondrial‐DNA data suggest that the distance between islands is larger than between color morphs (A. J. Pérez‐Delgado, in litt. 2024).
3. *Camponotus suffusus bendigensis*: this Australian endemic subspecies mimics the model *Myrmecia fulvipes* . Correspondingly, it exhibits black body, red legs, and a golden gaster, while the nominate form has a reddish head and mesosoma (Merrill and Elgar 2000). According to the current taxonomic status (Bolton 2025), both taxa belong to the same species.
4. *Colobopsis imitans*: morphometric data indicate there is a single morphological cluster with three color morphs within *Co. imitans*, each mimicking different model species. One morph resembles *Co. truncata* in terms of color.

The Mediterranean *Cr. scutellaris* ‐group species, *Cr. scutellaris*, *Cr. schmidti*, and *Cr. ionia* s. l., are unpalatable to lizards (H. C. Wagner 2025; Wagner et al. 2025) and are frequently mimicked by Camponotini ants (Seifert 2019; Schifani et al. 2022, 2024; Kraker and Wagner 2025; Wagner et al. 2025). These *Crematogaster* species share similar ecology, biology, nesting behavior, and aggressive tendencies with each other (Seifert 2018, Wagner et al. 2025); the aposematic coloration represents their most obvious differences (Borowiec and Salata 2025). Such distinct color differences among closely related ant species are unusual (cf. Seifert 2018). Therefore, it seems plausible that trail‐following mimics like *Ca. lateralis* and *Co. imitans*, which dilute the aposematic signal of the model and thereby increase its predation risk, drive the evolution of aposematism, color change, and maybe speciation in *Crematogaster*—which would be a case of the chase‐away hypothesis (Kikuchi and Pfennig 2013; Akcali et al. 2018; Kraker and Wagner 2025). While this hypothesis seems difficult to test empirically, our proposed scenario aligns—at least concerning the evolution of aposematism—with fundamental aposematism theory (Poulton 1890): Species which are of low profitability to be consumed by predators (because of, e.g., bad taste or toxicity) should evolve (visual) signals enabling predators to safely distinguish them from suitable prey species (Wallace 1889; Cott 1940; Ruxton et al. 2004).

### 4.4 Is *Do. quadripunctatus* the Ancestral Model?

A very intriguing thought experiment seeks to infer the ancestral model state of West‐Palearctic *Colobopsis* species from which the extant color morphs may have evolved. While the absence of comprehensive phylogenetic and paleontological data limits precise ancestral state reconstruction, we can offer insights based on coloration and biogeography. Our hypothesis centers on a subtle yet distinctive character found across *Colobopsis* species: the pair of white dots or the white stripe along the margin of the second gastral tergite.

These white markings contrast sharply with the surrounding dark gastral coloration and serve different functions across species. In the unpalatable *Do. quadripunctatus* , they represent aposematic signals (Figure 4), while in *Co. truncata* they function as Batesian mimicry (Forel 1874; H. C. Wagner 2019; Schifani et al. 2022). The presence of such markings in certain morphs of *Co. imitans*, which do not actively mimic *Do. quadripunctatus* (Schifani et al. 2022, 2024), is particularly noteworthy. Although these dots are less common and smaller than those in *Co. truncata* that use *Do. quadripunctatus* as the primary model, their presence in allopatry to *Do. quadripunctatus* suggests an ancestral state that once played a role in mimicry. This interpretation gains support from biogeographic evidence. Even on southern Mediterranean islands such as Crete and Cyprus, where *Do. quadripunctatus* is currently absent, rudimentary white gastral markings persist in local *Co. imitans* populations. This pattern suggests either that *Co. imitans* colonized these islands from northern populations where *Do. quadripunctatus* occurs, or that *Do. quadripunctatus* historically inhabited these islands before local extinction. In either scenario, the ancestors of contemporary blackish *Co. imitans* workers, now isolated from their original model, once coexisted with *Do. quadripunctatus*. Given that *Do. quadripunctatus* remains—besides *Colobopsis*—the only West‐Palearctic species exhibiting this distinctive whitish pattern (Lebas et al. 2016; Seifert 2018), evolutionary biologists familiar with these ant communities would likely concur that this mimetic trait evolved in sympatry with this model species.

### 4.5 Genetic Mechanisms Driving Mimicry in Ants

While the genetic mechanisms underlying mimicry are well characterized in butterflies—where wing pattern mimicry has been shown to involve tightly linked gene complexes or supergenes (Joron et al. 2011; Kunte et al. 2014)—the corresponding mechanisms in ants remain unexplored. One could wonder whether the observed polymorphic color‐mimicry patterns might also be controlled by any forms of genomic linkage that facilitate coordinated trait expression. Since color patterns in aposematic and mimetic ants are much simpler than in the colorful butterflies, we expect a mechanism in which only a few genes control the pigment production and its spatial expression across the main body regions (i.e., head, mesosoma, and gaster). The *Colobopsis* genome sequence has already been published (Vizueta et al. 2025); it could be a useful reference for establishing a data set to estimate divergence times among populations via population genetic analyses. Future studies might investigate the genomic basis of adaptive phenotypic evolution of mimicry in ants.

## 5 Conclusion

This study challenges the hypothesis that mimicry directly drives evolutionary divergence and serves as a precursor to speciation among mimetic ant species (Schifani et al. 2022). Although we demonstrated significant color differences in *Co. imitans* populations, we did not find taxonomically significant morphological variation in morphospace. This finding is in line with established principles in ant taxonomy, which considers coloration an unreliable marker for species delineation (Seifert 2018, 2019, 2025; Csősz et al. 2022). Specifically, three other mimetic camponotine ant species (Merrill and Elgar 2000; Seifert 2019; Pérez‐Delgado and Wagner 2024), as well as some butterfly (Mallet et al. 1998; Jiggins 2008; The Heliconius Genome Consortium 2012; Jones et al. 2013) and hoverfly species (Edmunds and Reader 2014), involve intraspecific entities without any clear signs of incipient speciation.

The widespread Mediterranean distribution of *Co. imitans*, coupled with its ability to maintain locally stable yet geographically variable color morphs that match regional model species, supports the view against rapid local speciation. Instead, our results suggest that *Co. imitans* populations have remained cohesive despite geographic separation. We can, however, not exclude that any ongoing speciation processes remain incomplete and have not yet resulted in morphologically distinguishable taxa.

Future research should focus on integrating molecular phylogenetic analyses with detailed ecological studies of model–mimic interactions across different geographic regions. Such approaches will help clarify whether the observed color variation represents ongoing gene flow and phenotypic microevolution, or reflects early stages of reproductive isolation that have not yet manifested in morphological divergence.

## Author Contributions

**Herbert C. Wagner:** conceptualization (equal), data curation (lead), formal analysis (equal), funding acquisition (lead), investigation (equal), methodology (equal), project administration (equal), resources (lead), software (supporting), validation (equal), visualization (equal), writing – original draft (equal), writing – review and editing (equal). **Sándor Csősz:** conceptualization (equal), data curation (supporting), formal analysis (equal), funding acquisition (supporting), investigation (equal), methodology (equal), project administration (equal), resources (supporting), software (lead), validation (equal), visualization (equal), writing – original draft (equal), writing – review and editing (equal).

## Funding

This research was funded in large part by the Austrian Science Fund (FWF) [10.55776/P35816] (on behalf of H.C.W.) and cofinanced by the HUN‐REN Hungarian Research Network and the National Research, Development, and Innovation Fund (Hungary) under Grant No. K 147781 (on behalf of S.C.). For the purpose of open access, the author has applied a CC BY public copyright license to any Author Accepted Manuscript version arising from this submission.

## Conflicts of Interest

The authors declare no conflicts of interest.

## Supporting information


**File S1:** Morphometric data of minor workers of the *Colobopsis truncata* complex in the West Palearctic.

**File S2:** R script to establish nest‐centroid clustering (Ward method), “part.kmeans,” and “part.hclust.”

## Data Availability

All data generated or analyzed during this study are included in this published article and its Supporting Information (available in the online version of this article at the publisher's website).
